# Janus nanofiber array pellicle: facile conjugate electrospinning construction, structure and bifunctionality of enhanced green fluorescence and adjustable magnetism

**DOI:** 10.1039/c8ra08588a

**Published:** 2018-12-21

**Authors:** Guoyi Wang, Qianli Ma, Jiao Tian, Libing Fan, Dan Li, Xiangting Dong, Wensheng Yu, Jinxian Wang, Guixia Liu

**Affiliations:** Key Laboratory of Applied Chemistry and Nanotechnology at Universities of Jilin Province, Changchun University of Science and Technology Changchun 130022 China dongxiangting888@163.com +86-0431-85383815 +86-0431-85582575

## Abstract

A [Fe_3_O_4_/polyvinyl pyrrolidone (PVP)]//[Tb(BA)_3_phen/PVP] Janus nanofiber array pellicle (denoted JNAP) was successfully constructed by facile conjugate electrospinning without twisting for the first time. The JNAP offers the dual-functionality of fluorescence and magnetism. This technology entirely solves the dilemma of the magnetic spinning dope and fluorescent spinning dope being easily mixed together during the parallel electrospinning process, as it achieves complete segregation of magnetic nanoparticles and fluorescent molecules. Moreover, conjugate electrospinning without twisting has fewer requirements on the viscosity of the spinning dope compared with parallel electrospinning, in which the two spinning dopes should have the same viscosity. It was satisfactorily found that the JNAP has higher fluorescence intensity than the corresponding non-aligned Janus nanofiber pellicle. The magnetism of the JNAP could be tailored by changing the doping amount of the Fe_3_O_4_ NPs. The JNAP has potential applications in nanotechnology and biomedicine, *etc.*, due to its enhanced green fluorescence and adjustable magnetism. In addition, this design concept and manufacturing process provide a facile way for preparing other one-dimensional Janus nanomaterials with multifunctionality.

## Abstract 2

A [Fe_3_O_4_/PVP]//[Tb(BA)_3_phen/PVP] Janus nanofiber array pellicle with enhanced green fluorescence and adjustable magnetism dual-functionality was constructed *via* facile conjugate electrospinning.

## Graphical abstract

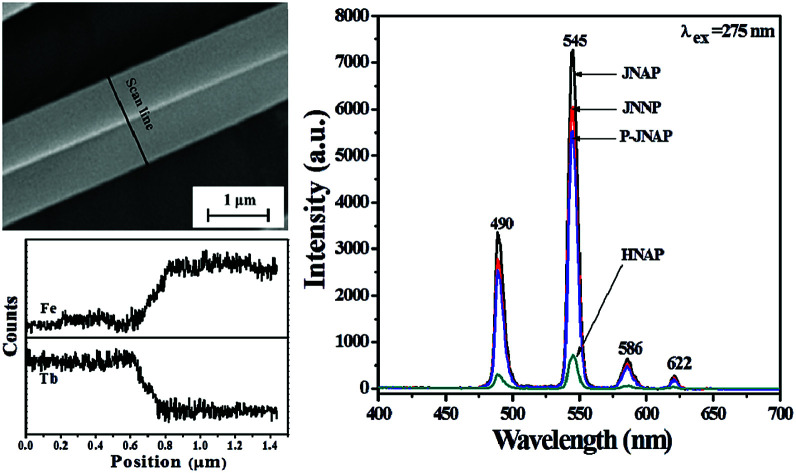

## Introduction

It is becoming harder to meet the needs of many emerging practical technologies using single-functional materials alone. Therefore, research into the preparation and performance of multifunctional materials has become increasingly important in the realm of materials science and technology.^1–3^ Fluorescent-magnetic bifunctional nanomaterials have attracted much attention of researchers due to their potential uses in cell imaging, cancer studies, sensing and biomolecular detection, *etc.*^4–9^ For instance, fluorescent-magnetic bifunctional nanomaterials are ideal candidates for drug delivery because the loaded drug can be transported to a specific location using magnetic navigation, and meanwhile, the real-time position of the drug can be monitored by measuring fluorescence signals emitted from the fluorescent-magnetic bifunctional nanomaterial.^10,11^ As another example, fluorescent-magnetic bifunctional nanomaterials possess the property of dual-mode imaging, including fluorescent imaging and magnetic resonance imaging, which is beneficial for biological detection.^12,13^ However, researchers in this field must face the plight that if the magnetic nanoparticles and fluorescent molecules are directly mixed, the fluorescence intensity of the sample will be greatly reduced.^14,15^ Hence, exceptional structures need to be designed and constructed to separate magnetic nanoparticles from fluorescent molecules in an integrated system. Inspired by Janus, a god in Roman mythology, an exceptive “Janus structure” has been proposed by several research groups.^16,17^ Researchers have proposed that magnetic nanoparticles and fluorescent molecules should be respectively added to both sides of a Janus material in order to isolate two different substances from each other and reduce the detrimental influence of magnetic nanoparticles on the fluorescence intensity.^18,19^ Magnetic-fluorescent bifunctional Janus nanofibers are typical Janus materials that realize separation of magnetic nanoparticles from fluorescent molecules. According to the literature, Janus nanofibers can be gained by parallel electrospinning, where two kinds of spinning dopes are respectively loaded into two syringes to generate magnetic-fluorescent bifunctional Janus nanofibers using a specially-made parallel spinneret under electrospinning.^20–29^ Xi, *et al.*^30,31^ fabricated flexible magnetic-photoluminescent bifunctional Janus nanofibers by parallel electrospinning. It has been proven that such Janus nanofibers have stronger fluorescence intensity than the counterpart composite nanofibers. However, parallel electrospinning still has a drawback in that it is difficult to fulfill complete segregation of the magnetic nanoparticles and the fluorescent molecules, since the two spinning dopes are easily mixed at the outlet of spinneret.^32^ Consequently, it is a pressing subject of study to develop a new technique to overcome this drawback.

Conjugate electrospinning is an excellent technique for constructing one-dimensional nanomaterials. This technique can be divided into two kinds: conjugate electrospinning with twisting^33–35^ and that without twisting.^36,37^ Conjugate electrospinning with twisting is used to prepare nanofiber yarns which can be applied to tissue repair, nerve regeneration and electrically conductive material, *etc.*^38–40^ Recently, this method has been utilized to construct magnetic-fluorescent bifunctional nanofiber yarns. Fan, *et al.* used conjugate electrospinning with twisting to fabricate heterogeneous nanofiber yarns to effectively separate magnetic nanoparticles from fluorescent molecules.^41,42^ To date, there are only a few studies on conjugate electrospinning without twisting, which mainly focus on constructing materials for photocatalysis, and those with mechanical and waterproof properties.^43–45^ A magnetic-fluorescent bifunctional Janus nanofiber array pellicle built by conjugate electrospinning without twisting has not been reported.

In this work, polyvinyl pyrrolidone (PVP), Fe_3_O_4_ nanoparticles (NPs) and Tb(BA)_3_phen were respectively used as a template, magnetic material and fluorescent compound. Tb(BA)_3_phen possesses excellent fluorescence properties due to the unique f–f transition of Tb^3+^. It has become one of the most important fluorescent materials at present. Fe_3_O_4_ NPs can be widely used in many fields, such as targeted therapy, magnetically-controlled switches, electronics and biological processes due to their unique superparamagnetism, good biocompatibility and high permeability. A [Fe_3_O_4_/PVP]//[Tb(BA)_3_phen/PVP] Janus nanofiber array pellicle (abbreviated as JNAP) with magnetic-fluorescent bifunctionality was constructed by conjugate electrospinning without twisting (called conjugate electrospinning hereinafter). To highlight the excellent performance of the JNAP, a series of comparative samples were also constructed by conjugate electrospinning and parallel electrospinning. Finally, the as-prepared samples were systematically characterized using the relevant test instruments, and several new findings were obtained.

## Experimental

### Chemicals

Tb_4_O_7_ (99.99%), concentrated nitric acid (HNO_3_), 1,10-phenanthroline (phen), benzoic acid (BA), FeSO_4_·7H_2_O, FeCl_3_·6H_2_O, polyethylene glycol (PEG, *M*_w_ ≈ 20 000), NH_4_NO_3_, ammonia (NH_3_·H_2_O), oleic acid (OA), polyvinyl pyrrolidone (PVP) and ethanol (CH_3_CH_2_OH) were used to prepare the samples. All chemicals were of analytical grade.

### Syntheses of OA-modified Fe_3_O_4_ NPs and terbium complexes

OA-modified Fe_3_O_4_ NPs (denoted as Fe_3_O_4_ NPs for short) and Tb(BA)_3_phen were prepared according to the literature.^46,47^

### Electrospinning process

Two different types of spinning dopes (named spinning dope A and spinning dope B) were used to prepare the Janus nanofibers. Spinning dope A, with fluorescence properties, was prepared as follows: PVP (1 g) was fully dissolved in ethanol (7 g) under magnetic stirring, and then a certain amount of Tb(BA)_3_phen complex was uniformly dispersed in the solution to form spinning dope A. Spinning dope B, with magnetic properties, was fabricated as follows: Fe_3_O_4_ NPs were dispersed in ethanol (7 g) under ultrasonication for 20 min, and then PVP (1 g) was added into the above suspension under mechanical stirring. The actual ingredients of spinning dope A and B are respectively shown in Tables 1 and 2.

**Table 1:** Ingredients of spinning dope A

Spinning dope A	Tb(BA)_3_phen : PVP (wt%)	Tb(BA)_3_phen (g)	PVP (g)	CH_3_CH_2_OH (g)
S_A1_	5%	0.0500	1.0000	7.0000
S_A2_	10%	0.1000	1.0000	7.0000
S_A3_	15%	0.1500	1.0000	7.0000
S_A4_	20%	0.2000	1.0000	7.0000
S_A5_	25%	0.2500	1.0000	7.0000

**Table 2:** Ingredients of spinning dope B

Spinning dope B	Fe_3_O_4_ NPs/PVP (mass ratio)	Fe_3_O_4_ NPs (g)	PVP (g)	CH_3_CH_2_OH (g)
S_B1_	0.5 : 1	0.5000	1.0000	7.0000
S_B2_	1 : 1	1.0000	1.0000	7.0000
S_B3_	1.5 : 1	1.5000	1.0000	7.0000
S_B4_	2 : 1	2.0000	1.0000	7.0000

A device diagram for constructing the [Fe_3_O_4_/PVP]//[Tb(BA)_3_phen/PVP] JNAP by conjugate electrospinning is presented in Table 3. The spinning dopes A and B were separately loaded into two syringes with plastic spinnerets. The angle between the syringe and the horizontal line was *ca.* 45° in the conjugate electrospinning device. To obtain the array pellicle, a rotating drum was used as a collector. The corresponding spinning dopes and detailed spinning conditions are summarized in Table 3.

**Table 3:** Electrospinning device diagrams, corresponding spinning dopes and electrospinning conditions of samples

Samples	Electrospinning device diagrams	Spinning dopes	Electrospinning conditions
JNAP	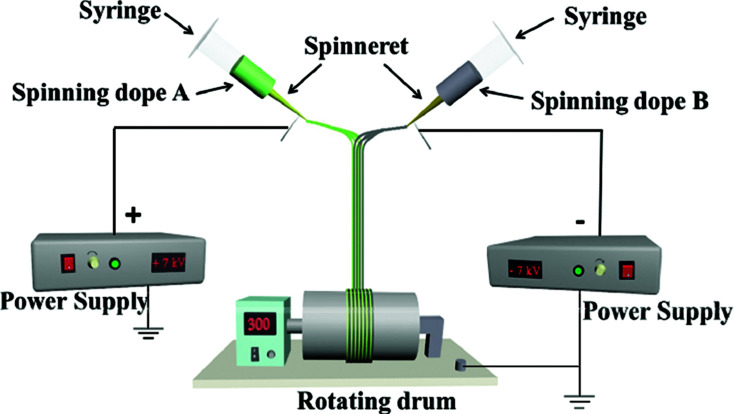	Spinning dope A: S_A1_, S_A2_, S_A3_, S_A4_ and S_A 5_, Spinning dope B: S_B1_	Collector: rotating drum (10 cm in diameter), rotation speed: 300 rpm, distance between the two spinnerets: 15 cm, curing distance: 25 cm, applied voltages: ± 7 kV, temperature: 15 ± 5 °C, relative humidity: 25 ± 5%
JNNP	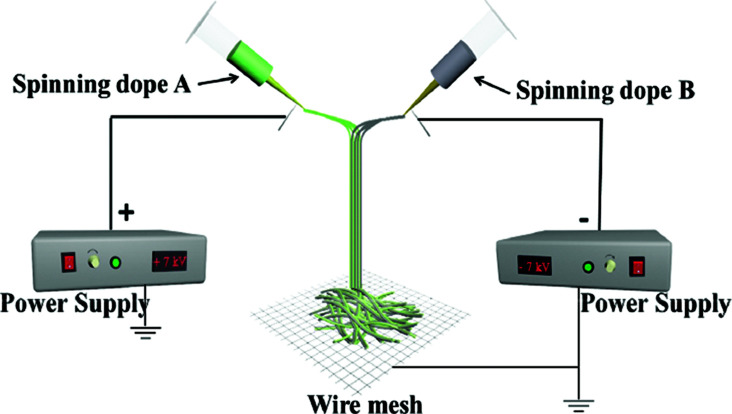	Spinning dope A: S_A3_, Spinning dope B: S_B1_	Collector: wire mesh, distance between the two spinnerets: 15 cm, curing distance: 25 cm, applied voltages: ± 7 kV, temperature: 15 ± 5 °C, relative humidity: 25 ± 5%
P-JNAP	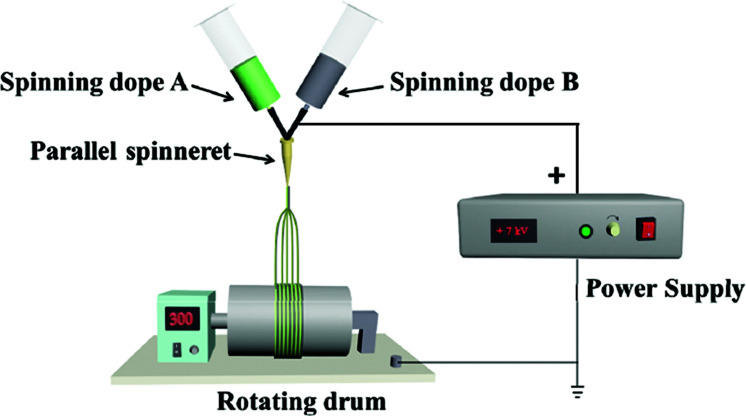	Spinning dope A: S_A3_, Spinning dope B: S_B1_	Collector: rotating drum (10 cm in diameter), rotation speed: 300 rpm, curing distance: 25 cm, applied voltages: + 7 kV, temperature: 15 ± 5 °C, relative humidity: 25 ± 5%
HNAP	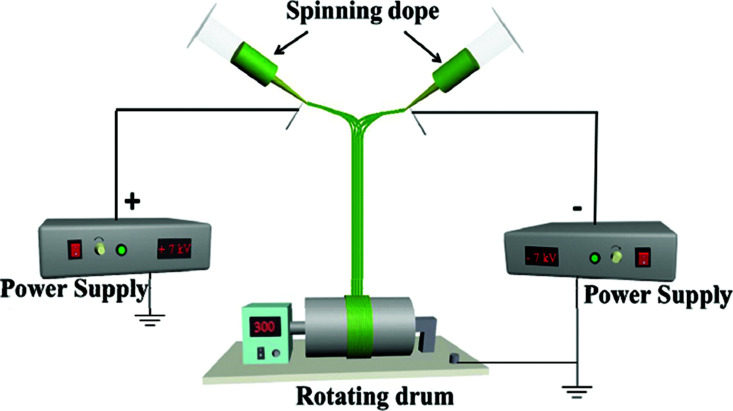	Spinning dope: mixing isopyknic S_A3_ and S_B1_	Collector: rotating drum (10 cm in diameter), rotation speed: 300 rpm, distance between the two spinnerets: 15 cm, curing distance: 25 cm, applied voltages: ± 7 kV, temperature: 15 ± 5 °C, relative humidity: 25 ± 5%

For comparison, a [Fe_3_O_4_/PVP]//[Tb(BA)_3_phen/PVP] Janus nanofiber non-array pellicle (referred to as JNNP) was prepared by conjugate electrospinning with a wire mesh as the collector, and a parallel electrospinning-made [Fe_3_O_4_/PVP]//[Tb(BA)_3_phen/PVP] Janus nanofiber array pellicle (named P-JNAP) was constructed by using spinning dopes S_A3_ and S_B1_. Another spinning dope was prepared by evenly mixing equal volumes of spinning dopes S_A3_ and S_B1_, and then a [Fe_3_O_4_/Tb(BA)_3_phen/PVP] homogeneous nanofiber array pellicle (called HNAP) was also fabricated by conjugate electrospinning. The device diagrams and specific conditions for the comparative samples are also displayed in Table 3.

### Characterization methods

The phase compositions of Fe_3_O_4_ NPs, JNAP, JNNP, HNAP and P-JNAP were analyzed using X-ray power diffraction (XRD) with Cu Kα radiation. The operation voltage and current were respectively kept at 40 kV and 20 mA. The morphology of the JNAP was observed using a field-emission scanning electron microscope (FESEM), equipped with energy-dispersive X-ray spectroscopy (EDS). A Hitachi fluorescence spectrophotometer F-7000 was used to investigate the fluorescence of different samples when the excitation and emission slits were 2.5 nm and 2.5 nm. The magnetic properties of the samples were measured using a vibrating sample magnetometer (VSM).

## Results and discussion

### Phase compositions

**Fig. 1:**
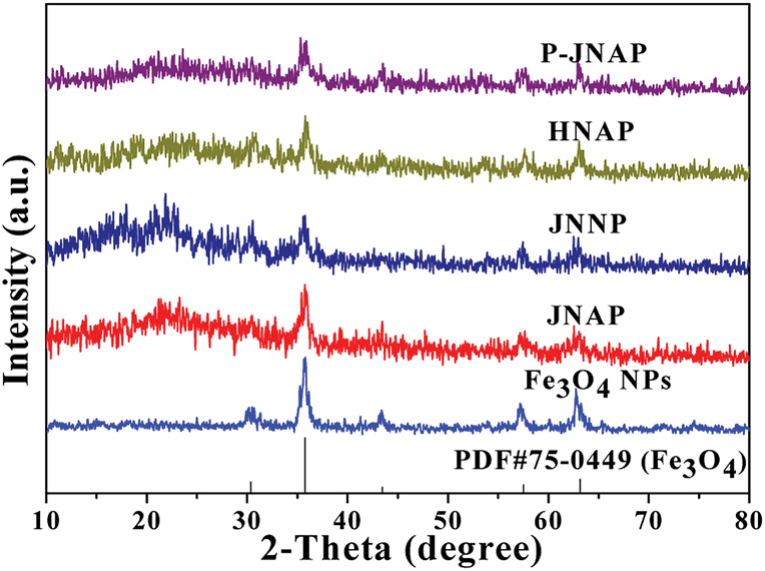
XRD patterns of the Fe_3_O_4_ NPs, JNAP, JNNP, HNAP and P-JNAP.

Fig. 1 exhibits the XRD patterns of the Fe_3_O_4_ NPs, JNAP, JNNP, HNAP and P-JNAP. The XRD patterns of the Fe_3_O_4_ NPs conform to the cubic structure of Fe_3_O_4_ (PDF#75-0499), and no characteristic peaks of other impurities are found. The corresponding diffraction peaks of Fe_3_O_4_ are also found in the four kinds of nanofiber pellicles, which indicates that the nanofiber pellicles contain Fe_3_O_4_ NPs. Meanwhile, the diffraction peak of the amorphous PVP (2*θ* ≈ 22°) can also be observed in the four kinds of nanofiber pellicles.

### Morphological and structural analyses

**Fig. 2:**
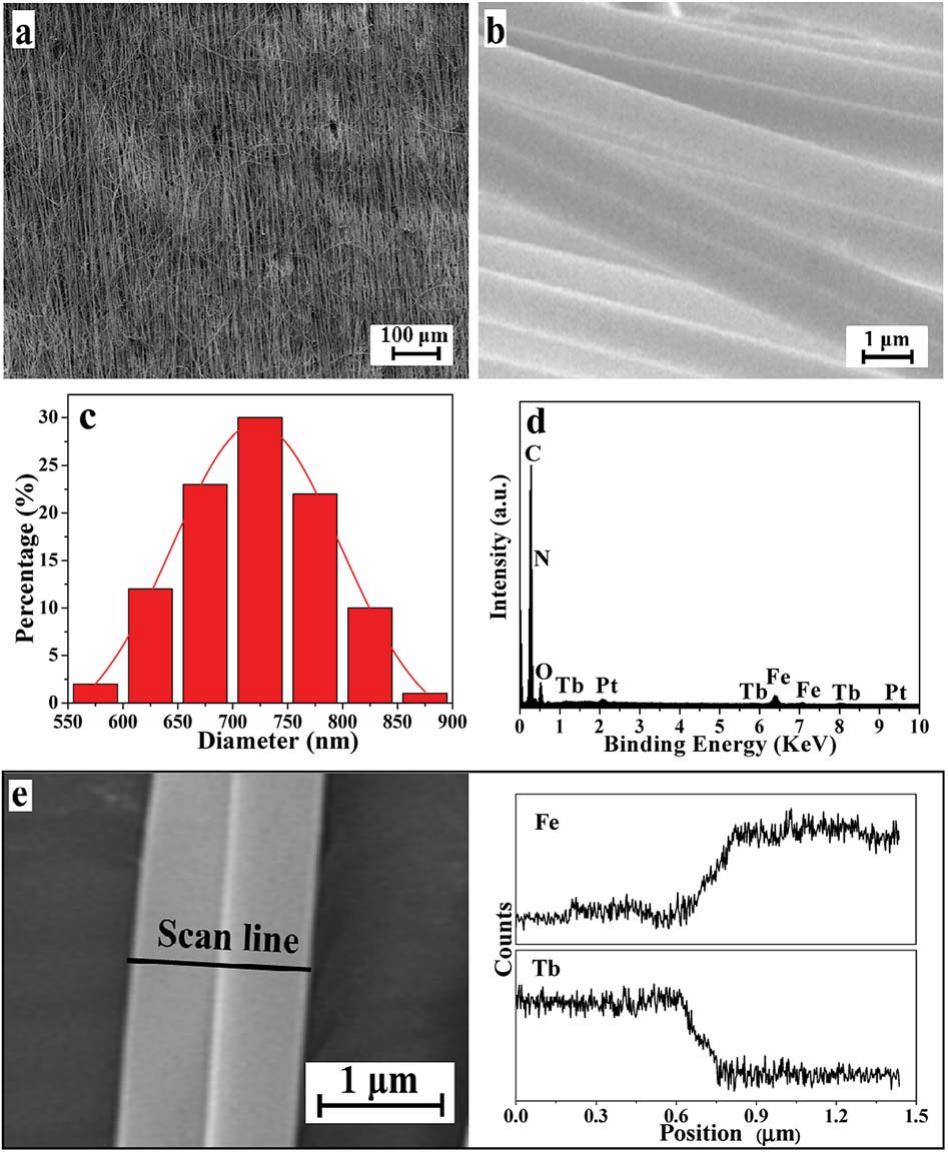
SEM images at low magnification (a) and high magnification (b) of the JNAP. Diameter distribution histogram of the nanofibers in Janus nanofibers in the JNAP (c). EDS spectrum of the JNAP (d). EDS line-scan analysis of a single Janus nanofiber in the JNAP (e).

SEM images, EDS spectra and diameter distribution histograms of the Janus nanofibers in JNAP are given in Fig. 2. As seen in Fig. 2a, the nanofibers are in good directional alignment and form an array pellicle. It can be seen in Fig. 2b that exceptive-structured Janus nanofibers are obtained, with every single Janus nanofiber being composed of two nanofibers bound together side-by-side. As shown in Fig. 2b and c, the diameter of each single nanofiber in the Janus nanofibers is almost equal (the average diameter of a single nanofiber is *ca.* 720 nm). The EDS spectrum of the JNAP (Fig. 2d) indicates that the Janus nanofibers are composed of the elements C, N, O, Fe, Tb and Pt. The Pt peak is attributed to the conductive film sprayed on the surface of the sample for SEM observation. Fig. 2e presents the EDS line-scan analysis results for the Janus nanofibers, where Fe and Tb elements indicate Fe_3_O_4_ and Tb(BA)_3_phen, respectively. The Fe and Tb elements are respectively found in two single nanofibers, further demonstrating that the Janus nanofibers were successfully constructed by conjugate electrospinning and the goal of segregating magnetic nanoparticles and fluorescent molecules has been realized.

### Fluorescence performance

**Fig. 3:**
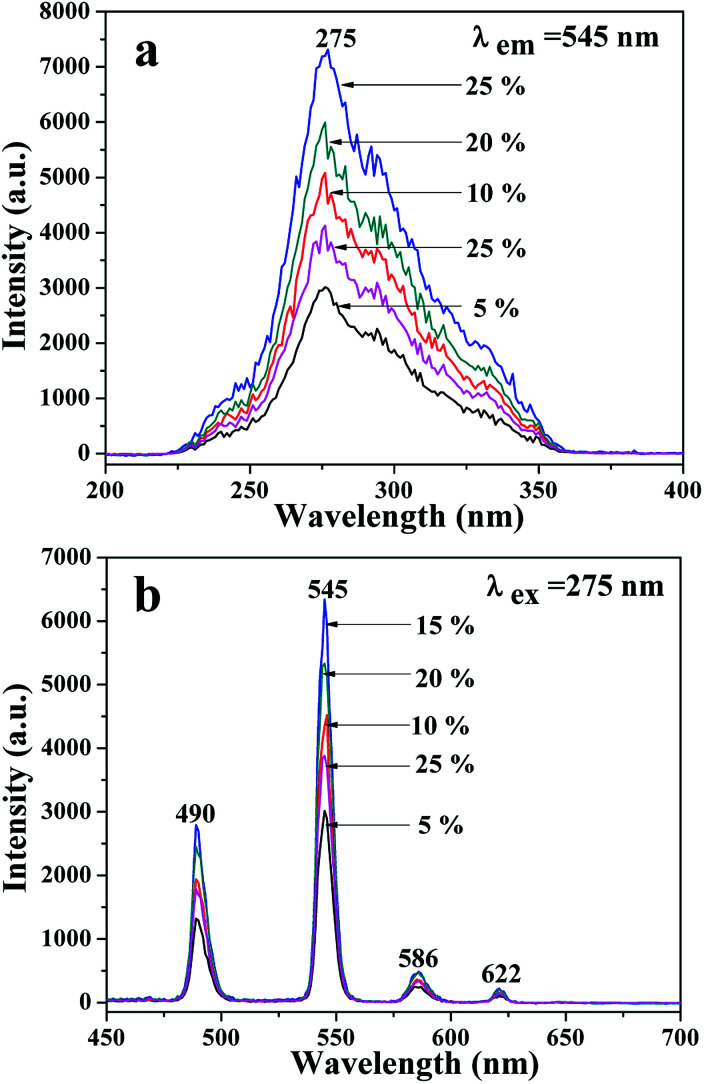
Excitation spectra (a) and emission spectra (b) of the JNAP samples doped with different amounts of Tb(BA)_3_phen when the mass ratio of Fe_3_O_4_ to PVP is 0.5 : 1.

A series of JNAP samples (the mass ratio of Fe_3_O_4_ NPs to PVP was 0.5 : 1, and the mass percentages of Tb(BA)_3_phen to PVP were 5%, 10%, 15%, 20% and 25%, respectively) were prepared to find the optimal mass percentage of Tb(BA)_3_phen to PVP. Fig. 3a and b respectively exhibit the excitation and emission spectra of the above-described JNAP samples. A broad excitation band from 200 nm to 400 nm of the JNAP was observed when the monitoring wavelength was 545 nm. The strongest excitation peak at 275 nm can be put down to the π → π* electron transition of the ligands. The strong characteristic emission peaks of Tb^3+^ were found at 490, 545, 586, 622 nm under 275 nm excitation. These peaks are attributed to the ^5^D_4_ → ^7^F_6_ (490 nm), ^5^D_4_ → ^7^F_5_ (545 nm), ^5^D_4_ → ^7^F_4_ (586 nm), ^5^D_4_ → ^7^F_3_ (622 nm) energy level transitions of Tb^3+^, respectively. The ^5^D_4_ → ^7^F_5_ transition at 545 nm (green light) is the predominant emission peak. The fluorescence intensity of the JNAP first enhances and then weakens with the increase of Tb(BA)_3_phen content, as revealed in Fig. 3. When the mass percentage of Tb(BA)_3_phen to PVP is 15%, the JNAP achieves the highest fluorescence intensity. When the mass percentage of Tb(BA)_3_phen to PVP exceeds 15%, the distribution of the Tb(BA)_3_phen becomes denser in the polymer matrix, resulting in stronger non-radiative transitions amongst the Tb^3+^ ions, which causes obvious reductions in the fluorescence intensity. Therefore, the optimum mass percentage of Tb(BA)_3_phen to PVP is 15%.

**Fig. 4:**
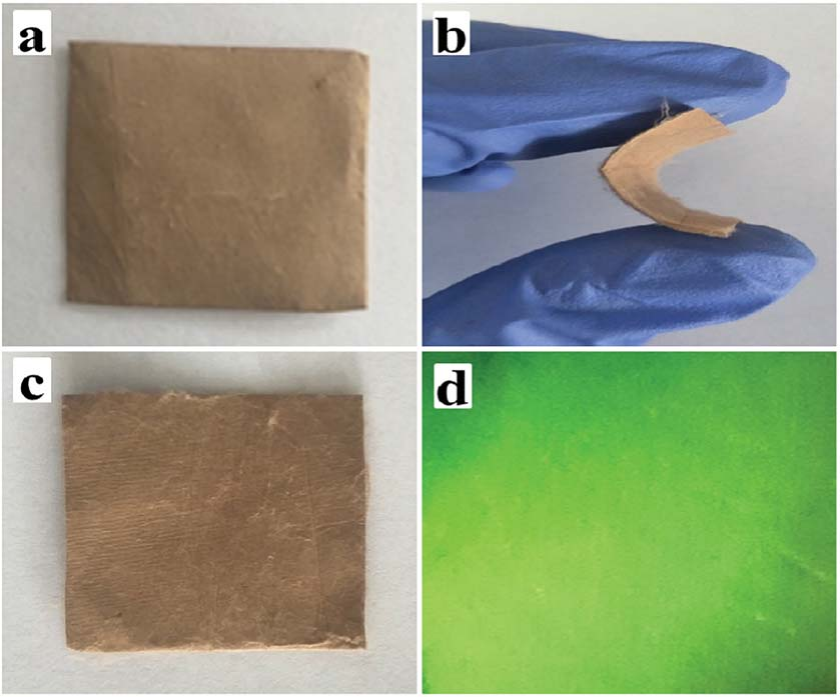
Physical digital photos of unbent JNAP (a), bent JNAP (b), recovered JNAP (c), and the JNAP under 275 nm UV illumination in darkness (d).

Physical photographs of the JNAP are displayed in Fig. 4. From Fig. 4a–c, it can be seen that the JNAP can be easily bent by hand and also possesses the ability of self-recovery, which proves that the JNAP is flexible. Fig. 4d shows a camera photograph of the JNAP under 275 nm UV illumination in darkness, indicating that the JNAP can emit green fluorescence.

**Fig. 5:**
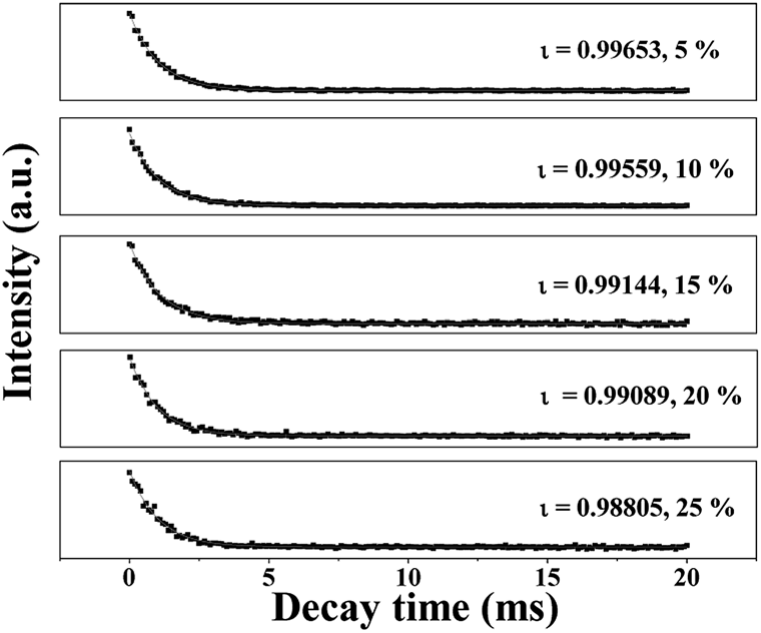
Fluorescence decay dynamics of the ^5^D_4_ → ^7^F_5_ transitions (*λ*_em_ = 545 nm) in the JNAP doped with varying amounts of Tb(BA)_3_phen.

The fluorescence decay curves of Tb^3+^ ions in the JNAP samples doped with different amounts of Tb(BA)_3_phen are shown in Fig. 5. The excitation wavelength is set to be 275 nm and the monitoring wavelength is 545 nm. It is generally known that the fluorescence decay curves follow the single-exponential decay:^48^*I*_*t*_ = *I*_0_ exp (−*t*/*ι*),where *I*_*t*_ signifies the intensity at time *t*, *I*_0_ represents the intensity at *t* = 0 and *ι* symbolizes the decay lifetime. The average lifetime values (*ι*/ms) of the JNAP are revealed in Fig. 5. As the content of Tb(BA)_3_phen increases, the fluorescence lifetime of the ^5^D_4_ → ^7^F_5_ (545 nm) transition is gradually decreased. The introduction of more Tb(BA)_3_phen leads to a reduced distance between Tb^3+^ ions in Tb(BA)_3_phen molecular clusters in the JNAP. Thus, the energy transfer among Tb^3+^ ions is increased, and the fluorescence lifetime of Tb^3+^ is shortened.^49^

**Fig. 6:**
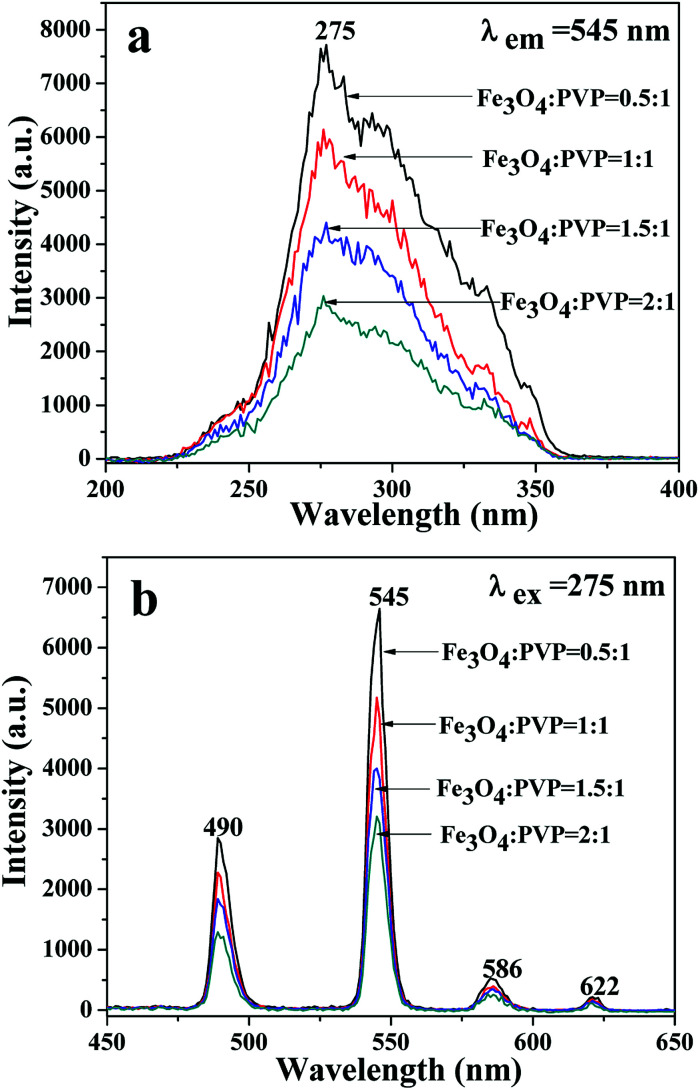
Excitation spectra (a) and emission spectra (b) of the JNAP doped with different amounts of Fe_3_O_4_ NPs when the mass percentage of Tb(BA)_3_phen to PVP is 15%.

**Fig. 7:**
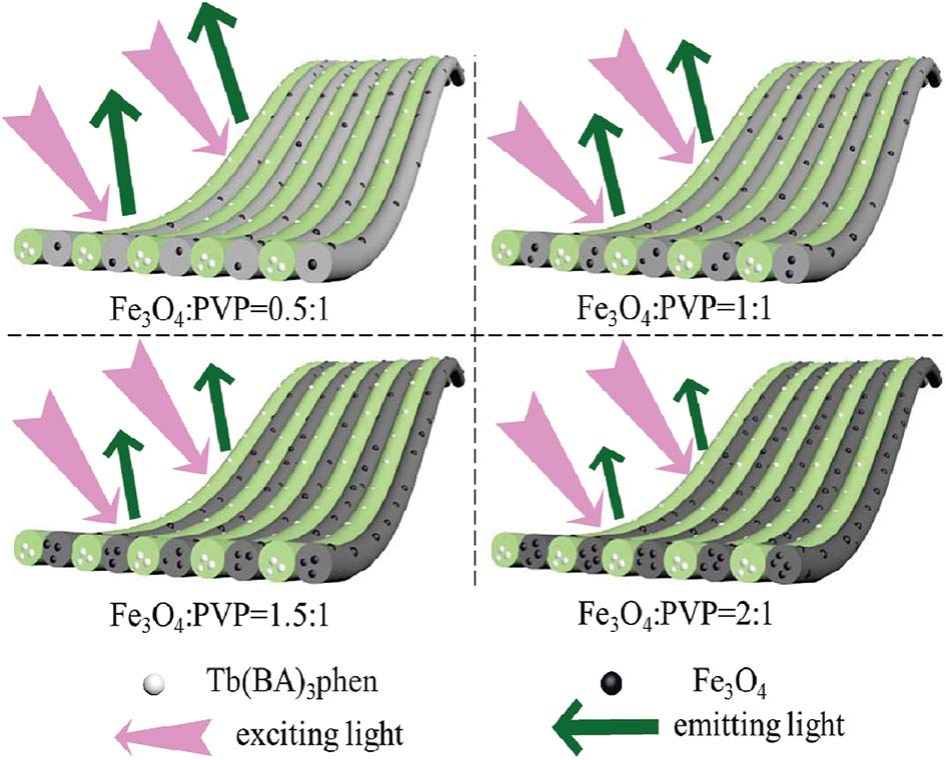
Schematic diagram of the excitation light and emission light in the JNAP doped with different amounts of Fe_3_O_4_ NPs when the mass percentage of Tb(BA)_3_phen to PVP is 15%.

A series of JNAP samples (the mass percentage of Tb(BA)_3_phen to PVP was 15% and the mass ratios of Fe_3_O_4_ NPs to PVP were 0.5 : 1, 1 : 1, 1.5 : 1, and 2 : 1, respectively) were prepared to explore the effect of different amounts of Fe_3_O_4_ NPs on the fluorescence intensity. The intensities of the excitation and emission peaks gradually decrease along with increasing content of Fe_3_O_4_ NPs added into the JNAP, as illustrated in Fig. 6. As previously reported, dark-colored Fe_3_O_4_ NPs can absorb visible light (400 nm < *λ* < 700 nm) and ultraviolet light (λ < 400 nm).^41^ Therefore, Fe_3_O_4_ NPs can absorb the excitation light and emission light of the JNAP, and the degree of absorption becomes stronger as the content of Fe_3_O_4_ NPs is increased. Moreover, the more Fe_3_O_4_ NPs added into the JNAP, the darker the color of the products and the more intense the absorption of excitation emission light by the Fe_3_O_4_ NPs, as revealed in Fig. 7, giving rise to lower fluorescence intensity.

**Fig. 8:**
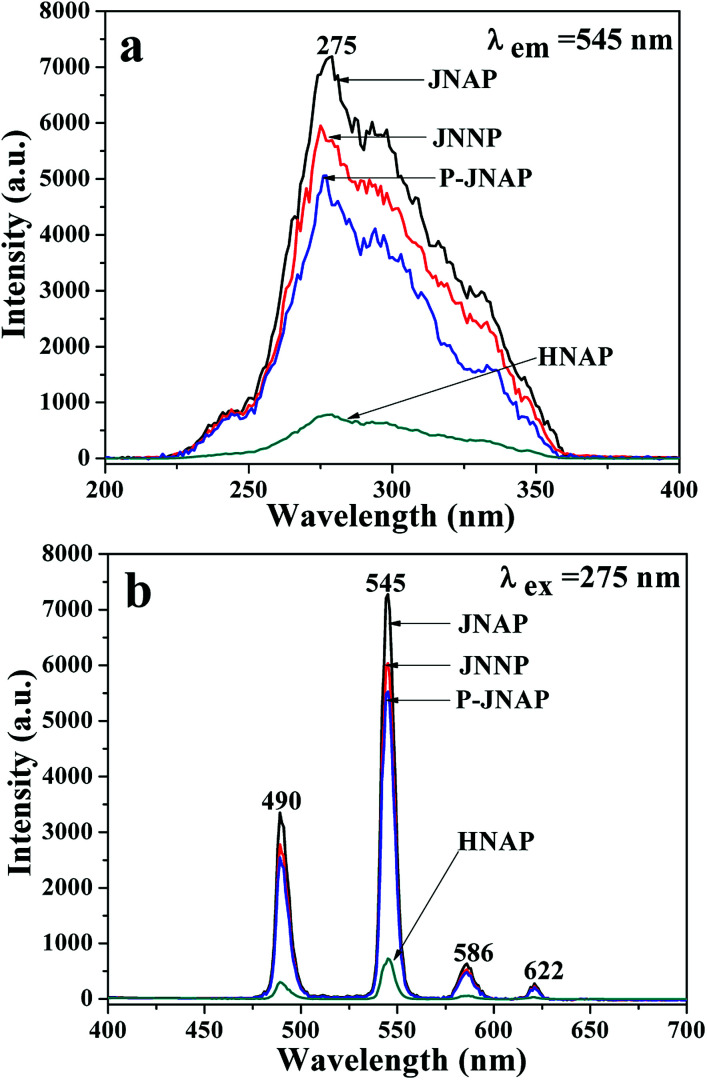
Excitation spectra (a) and emission spectra (b) of JNAP, JNNP, P-JNAP and HNAP.

**Fig. 9:**
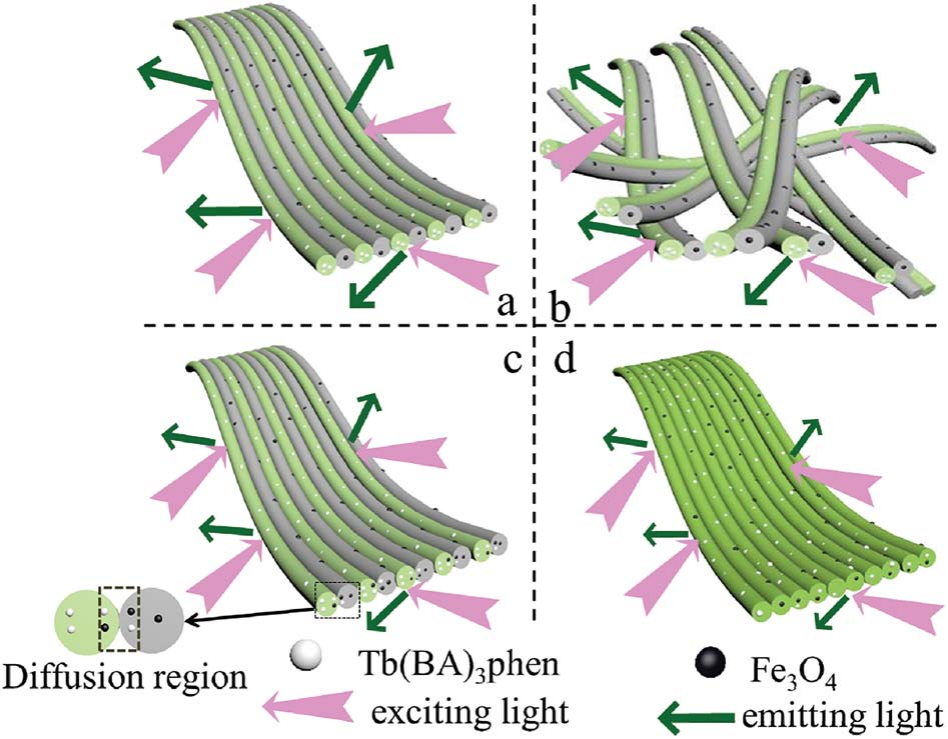
Schematic graphs of the excitation and emission light in JNAP (a), JNNP (b), P-JNAP (c) and HNAP (d).

Furthermore, the fluorescence intensities of samples with the same ingredients and content prepared by different methods were compared to highlight the superiority of the JNAP. The JNAP has the highest fluorescence intensity compared with three comparative samples. It has stronger fluorescence intensity than the P-JNAP, and HNAP possesses the lowest fluorescence intensity, as shown in Fig. 8. This outcome can be explained by three aspects: the special Janus structure, the arrangement of Janus nanofibers, and the electrospinning method. Fig. 9 presents schematic graphs of the excitation and emission light for the JNAP, JNNP, P-JNAP and HNAP. As seen from Fig. 9a, the JNAP is composed of aligned Janus nanofibers, and the Janus nanofibers are closely arranged. The Janus nanofibers in the upper layer can directly absorb excitation light and the produced emission light can be directly emitted without refraction or reflection, which is the most effective mode for fluorescence. Furthermore, the Fe_3_O_4_ NPs and Tb(BA)_3_phen molecules are absolutely segregated, leading to a reduced effect of the Fe_3_O_4_ NPs on the fluorescence performance. As depicted in Fig. 9b, the Fe_3_O_4_ NPs and Tb(BA)_3_phen molecules are also completely segregated in the JNNP. The surface of the JNNP is loose due to the unordered arrangement of Janus nanofibers. In this case, some of the excitation light can pass through the gaps in the upper layer and thereby excite the Janus nanofibers in lower layer. In this process, the Janus nanofibers in the upper layer can absorb some of the excitation light, and thus, the excitation light arriving to the Janus nanofibers in the lower layer is weakened. For the same reason, the emission light emitted from the lower layer is also absorbed by the upper layer in the JNNP. Hence, the fluorescence intensity of the JNNP is lower than that of the JNAP.

**Fig. 10:**
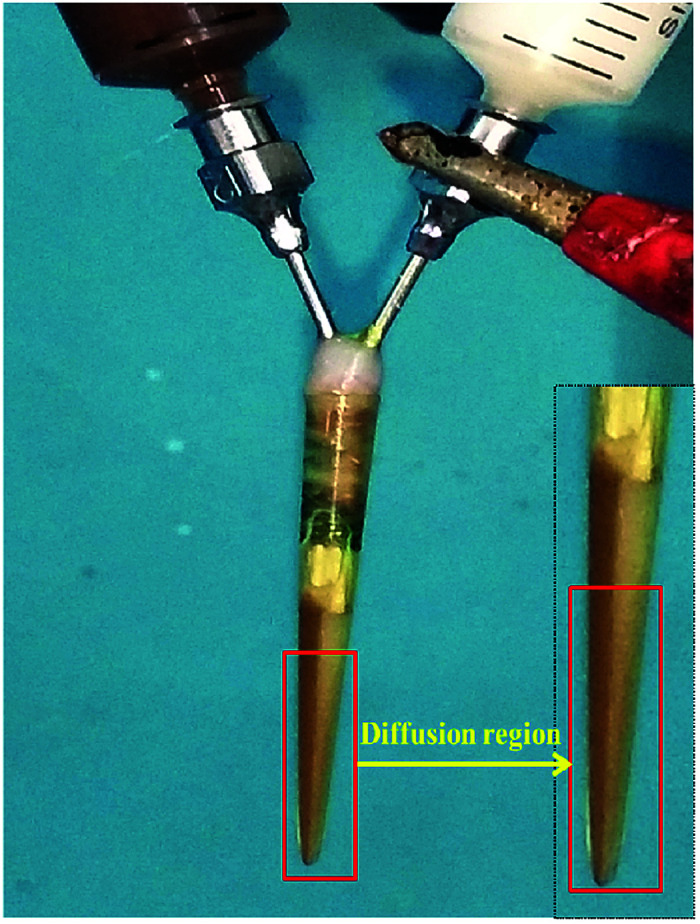
Digital photograph of parallel spinneret during parallel electrospinning process.

P-JNAP was prepared by parallel electrospinning. Fig. 10 describes the actual situation of two spinning dopes in parallel spinneret during the parallel electrospinning process. Inside the parallel spinneret, two spinning dopes mutually diffuse at the contact interfaces, so that diffusion regions exist at the contact interfaces between nanofibers in the Janus nanofibers. This results in an incomplete separation of Fe_3_O_4_ NPs from Tb(BA)_3_phen molecules in the Janus nanofibers. Thus, the fluorescence intensity of P-JNAP is lower than that of JNAP. As seen in Fig. 9d, the HNAP is totally made up of homogeneous nanofibers where Fe_3_O_4_ NPs and Tb(BA)_3_phen molecules are directly mixed, so that the fluorescence intensity of the HNAP is the lowest. The above new findings thoroughly prove that conjugate electrospinning has more advantages than parallel electrospinning in the fabrication of Janus nanofibers.

### Magnetic properties

**Fig. 11:**
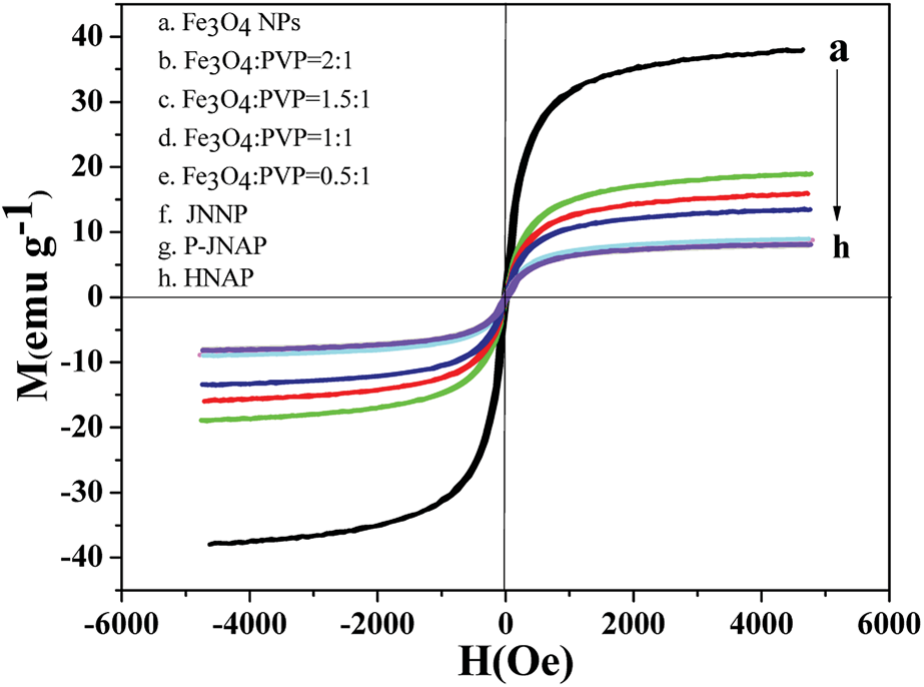
Hysteresis loops of the Fe_3_O_4_ NPs (a), the JNAP doped with different amounts of Fe_3_O_4_ NPs (b–e), JNNP (f), P-JNAP (g) and HNAP (h).

Typical hysteresis loops of the Fe_3_O_4_ NPs, the JNAP with different mass ratios of the Fe_3_O_4_ NPs and comparative samples are exhibited in Fig. 11, and corresponding saturation magnetization results are summarized in Table 4. It is known that the saturation magnetization of a magnetic compound material depends on the doping mass ratio of the magnetic material. As can be seen in Table 4, the saturation magnetization of the Fe_3_O_4_ NPs reaches 37.99 emu g^−1^. As the content of the Fe_3_O_4_ NPs increases in the JNAP, the saturation magnetization of the JNAP also increases from 8.93 to 18.99 emu g^−1^. Thus, the JNAP possesses adjustable magnetism *via* changing the doping amount of the Fe_3_O_4_ NPs. The magnetism of the comparative samples is similar to that of the JNAP (prepared by S_A3_/S_B1_) owing to the same content of Fe_3_O_4_ NPs in the samples.

**Table 4:** Saturation magnetization results of the Fe_3_O_4_ NPs, the JNAP doped with different amounts of Fe_3_O_4_ NPs and comparative samples

Samples	Saturation magnetization (emu g^−1^)
Fe_3_O_4_ NPs	37.99
**JNAP**	
Fe_3_O_4_ : PVP = 2 : 1	18.99
Fe_3_O_4_ : PVP = 1.5 : 1	15.86
Fe_3_O_4_ : PVP = 1 : 1	13.50
Fe_3_O_4_ : PVP = 0.5 : 1	8.93
**Comparative samples**	
JNNP	8.77
P-JNAP	8.07
HNAP	7.91

### Formation mechanism for the JNAP

**Fig. 12:**
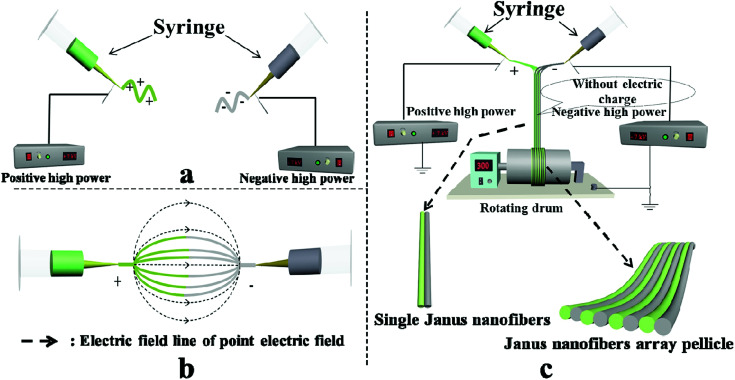
Schematic graph of the formation mechanism of Janus nanofibers and the JNAP: (a and c) front view of the device; (b) top view of the device.

Schematic graphs of the formation mechanism of Janus nanofibers and the JNAP are presented in Fig. 12. Under the effect of an electrostatic field, the spinning dopes in the two spinnerets form two bundles of continuous nanofibers with positive and negative charges after positive and negative power are applied to the two spinnerets, respectively, as seen in Fig. 12a. Here, an electric field is formed between the two spinnerets when positive and negative charges accumulate at the tip of the two spinnerets, and the directions of the electric field lines are indicated by the dotted line (from the positive pole to the negative pole), as shown in Fig. 12b. Magnetic nanofibers and fluorescent nanofibers attract each other along the path of the electric field. After the two kinds of nanofiber bundles meet in the middle of the two spinnerets, the electrical charges in the two kinds of nanofiber bundles are neutralized, and the formed Janus nanofibers move downward by gravity. Due to electrostatic repulsion, the same nanofibers (*e.g.* magnetic nanofiber and fluorescent nanofiber) cannot get together to form a Janus nanofiber in this process. Therefore, [Fe_3_O_4_/PVP]//[Tb(BA)_3_phen/PVP] Janus nanofibers are obtained. Fig. 12c describes the process of collecting an electrically neutral Janus nanofiber bundle with the rotating drum. At this point, the Janus nanofibers are affected by the vortex generated by the rotating drum in the experimental environment, so that the Janus nanofibers shake slightly and are drawn to and wrap around the rotating drum to form the JNAP.

## Conclusions

In summary, a [Fe_3_O_4_/PVP]//[Tb(BA)_3_phen/PVP] magnetic-fluorescent bifunctional Janus nanofiber array pellicle was successfully fabricated *via* facile conjugate electrospinning with a rotating drum as a collection device. The JNAP is made up of aligned Janus nanofibers. Each Janus nanofiber is composed of a magnetic [Fe_3_O_4_/PVP] nanofiber and a fluorescent [Tb(BA)_3_phen/PVP] nanofiber. The diameter of each single nanofiber in the Janus nanofibers is *ca.* 720 nm. It is noteworthy that the magnetism of the JNAP can be adjusted by adding diverse amounts of Fe_3_O_4_ NPs and the JNAP can emit green light under the excitation of ultraviolet light. Compared with three comparative samples, the JNAP has the highest fluorescence intensity owing to its peculiar Janus structure, which achieves effective segregation of Fe_3_O_4_ NPs and Tb(BA)_3_phen molecules, as well as directional alignment of the Janus nanofibers. More importantly, conjugate electrospinning can be extended to manufacture other Janus nanofibers with multifunctionality.

## Conflicts of interest

There are no conflicts of interest to declare.

## Supplementary Material
